# Iron-Bound Lipocalin-2 from Tumor-Associated Macrophages Drives Breast Cancer Progression Independent of Ferroportin

**DOI:** 10.3390/metabo11030180

**Published:** 2021-03-19

**Authors:** Christina Mertens, Matthias Schnetz, Claudia Rehwald, Stephan Grein, Eiman Elwakeel, Andreas Weigert, Bernhard Brüne, Michaela Jung

**Affiliations:** 1 Institute of Biochemistry I, Faculty of Medicine, Goethe-University Frankfurt, Theodor-Stern-Kai 7, 60590 Frankfurt am Main, Germany; Christina.Mertens@med.uni-heidelberg.de (C.M.); matthias.schnetz@t-online.de (M.S.); c.rehwald@ymail.com (C.R.); elwakeel@biochem.uni-frankfurt.de (E.E.); weigert@biochem.uni-frankfurt.de (A.W.); b.bruene@biochem.uni-frankfurt.de (B.B.); 2 Department of Mathematics, Temple University, Philadelphia, PA 19122, USA; grein@temple.edu; 3 Frankfurt Cancer Institute, Goethe-University Frankfurt, 60596 Frankfurt, Germany; 4 Project Group Translational Medicine and Pharmacology TMP, Fraunhofer Institute for Molecular Biology and Applied Ecology, 60596 Frankfurt am Main, Germany

**Keywords:** tumor-associated macrophages (TAM), iron, ferroportin, lipocalin-2, iron-trafficking, tumor microenvironment, tumor stroma

## Abstract

Macrophages supply iron to the breast tumor microenvironment by enforced secretion of lipocalin-2 (Lcn-2)-bound iron as well as the increased expression of the iron exporter ferroportin (FPN). We aimed at identifying the contribution of each pathway in supplying iron for the growing tumor, thereby fostering tumor progression. Analyzing the expression profiles of Lcn-2 and FPN using the spontaneous polyoma-middle-T oncogene (PyMT) breast cancer model as well as mining publicly available TCGA (The Cancer Genome Atlas) and GEO Series(GSE) datasets from the Gene Expression Omnibus database (GEO), we found no association between tumor parameters and Lcn-2 or FPN. However, stromal/macrophage-expression of Lcn-2 correlated with tumor onset, lung metastases, and recurrence, whereas FPN did not. While the total iron amount in wildtype and Lcn-2^−/−^ PyMT tumors showed no difference, we observed that tumor-associated macrophages from Lcn-2^−/−^ compared to wildtype tumors stored more iron. In contrast, Lcn-2^−/−^ tumor cells accumulated less iron than their wildtype counterparts, translating into a low migratory and proliferative capacity of Lcn-2^−/−^ tumor cells in a 3D tumor spheroid model in vitro. Our data suggest a pivotal role of Lcn-2 in tumor iron-management, affecting tumor growth. This study underscores the role of iron for tumor progression and the need for a better understanding of iron-targeted therapy approaches.

## 1. Introduction

The tumor microenvironment (TME) of solid, e.g., breast tumors, generally consists of diverse cell types, including neoplastic cells and recruited or resident immune cells in the stroma [1,2]. This highly heterogeneous nature of the TME is tightly linked to numerous hallmarks of cancer, including metabolic adaptations, among them a disordered iron metabolism [3]. Within the population of immune cells, macrophages (MΦ) play a major role and might comprise up to 50% of the TME in breast cancer [4]. MΦ are a versatile and functionally heterogeneous cell population. In tumors, a diverse set of MΦ subpopulations can be found, which is determined by local microenvironmental cues. The heterogeneity of the different MΦ phenotypes in the TME comprises the localization within the tumor and the interplay of MΦ with other cells [5,6,7]. The crosstalk of MΦ and neoplastic cells especially involve a complex network of cytokines, chemokines, exosomes as well as metabolites, including iron [8,9,10,11]. Our previous studies provide evidence that MΦ cells acquire an iron-release phenotype in both mammary [12] and renal [13] cancer. We concluded that MΦ may serve as an important source of iron to fuel the elevated iron demand of neoplastic cells, which, in turn, fosters tumor growth and progression. At the molecular level, iron-releasing MΦ express enhanced levels of the iron exporter ferroportin (FPN) as well as the iron-transporter lipocalin-2 (Lcn-2), both adding to iron secretion to the TME.

FPN is a transmembrane protein and the only so far known exporter for non-heme iron in mammalian cells. Due to this unique function, its expression is controlled in multiple ways, ranging from transcriptional control, i.e., by nuclear factor (erythroid-derived 2)-like 2 (Nrf2) [14], post-transcriptional regulation through the iron-regulatory protein/iron-responsive element (IRP/IRE) system [15], as well as post-translationally via the peptide hormone hepcidin [16]. Previous studies reported FPN mutations and polymorphisms in relation to inflammation-associated pathological conditions [17] as well as in cancer [18]. FPN down-regulation in cancer cells enhanced tumor growth and metastasis, both in patients [19] as well as in experimental tumor models. Moreover, we recently proved enhanced FPN expression in tumor-associated macrophages (TAM) in renal cancer [13]. However, we did not observe any correlation with tumor stage or grade, suggesting that FPN expression is not a crucial feature of the TAM iron-release phenotype. 

Another iron transport protein that was described to play a crucial role in tumor development is Lcn-2, which belongs to the lipocalin superfamily of carrier proteins [20,21]. The unique characteristic of Lcn-2 is based on its extraordinarily high affinity for bacterial siderophores, which accounts for its pivotal role in innate immune responses [22]. Lcn-2 not only serves as a bacteriostatic agent secreted from MΦ during infection but is also being produced and released from TAM within the TME, with profound pro-tumor characteristics. MΦ-derived Lcn-2 fosters mammary tumor growth and proliferation [12,23,24] as well as metastasis through the induction of epithelial-to-mesenchymal transition (EMT) [24] and tumor lymphangiogenesis [23,25]. At the molecular level, Lcn-2 is regulated through the activation of the signal transducer and activator of transcription 3 (STAT3) and CCAAT-enhancer-binding protein (C/EBP) β transcription factors in MΦ in response to the uptake and recycling of apoptotic tumor cells [23,26]. Recently, we showed that the iron-load crucially determined the biological function of Lcn-2. Iron-loaded Lcn-2 contributed to pro-tumor functions, whereas iron-free Lcn-2 had adverse effects in renal cancer [27]. 

Thus, given the importance of iron-export and iron-transporting proteins such as FPN and Lcn-2 in the tumor context, we aimed at clarifying their role and/or interplay in breast cancer. In the present study, we provide evidence that both FPN and Lcn-2 mRNA expression analyzed in whole tissue samples is not regulated in breast cancer, examined by TCGA data base analysis as well as in the experimental PyMT spontaneous murine breast cancer model. However, MΦ-expressed FPN and Lcn-2 are significantly elevated in tumor tissue compared to healthy breast tissue, but only Lcn-2 was significantly associated with tumor onset, metastasis as well as recurrence. We further identified iron-loaded Lcn-2 in extracellular tumor fluids, which, in turn, favored tumor proliferation, matrix adhesion, and migration of tumor cells in vitro.

## 2. Results

### 2.1. Enhanced Iron Amount in Mammary Tumor Tissue Is Associated with Poor Outcome

**Figure 1:**
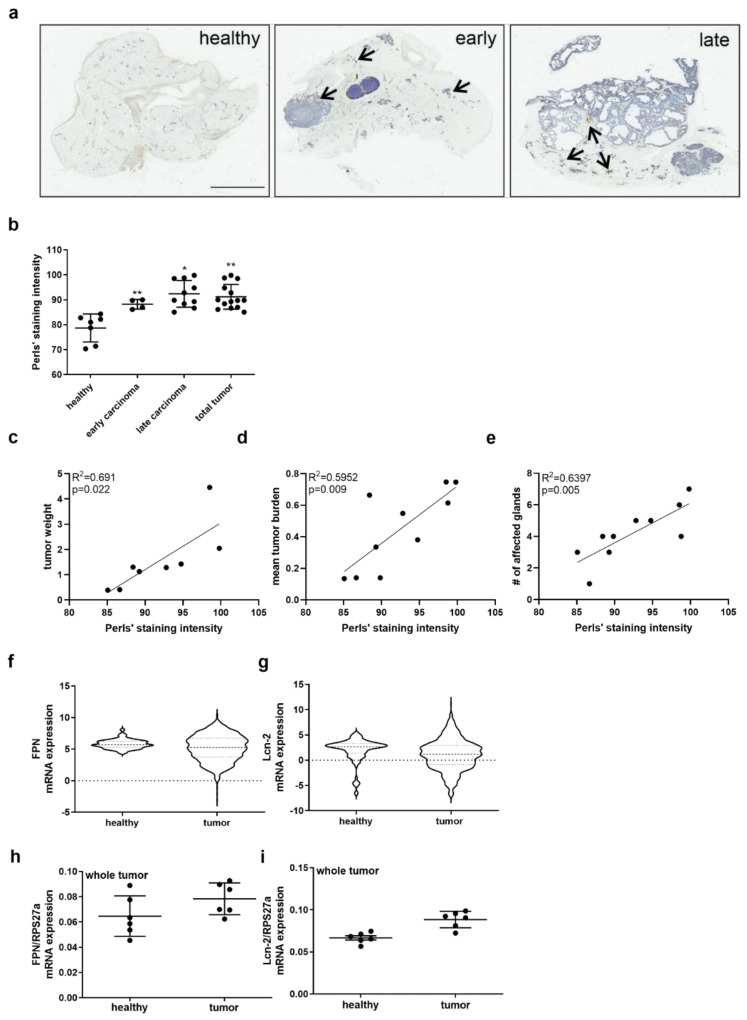
Analysis of the tumor iron metabolism. (**a**) Perls’ stain (blue colored) to visualize iron in murine healthy, early and late carcinoma tissue. Arrows indicate hemosiderin deposits (brown colored). Representative pictures of six mice. Scale bar: 5 mm (**b**) Quantification of stained iron amount in tissue sections by ImageJ. (**c**,**d**,**e**) Correlation of the tumor iron amount to tumor parameters: (**c**) tumor weight, (**d**) tumor burden, and (**e**) the number of affected mammary glands. (**f**,**g**) Analysis of TCGA-BRCA data sets from breast cancer compared to healthy tissue for the mRNA expression of (**f**) FPN and (**g**) Lcn-2 (n = 110). (**h**,**i**) Quantification of the mRNA expression of (**h**) FPN and (**i**) Lcn-2 in whole tumor homogenates from wildtype PyMT mice compared to murine healthy breast tissue. Graphs are displayed as means ± SEM. Statistically significant differences were calculated after analysis of variance (ANOVA) and Bonferroni’s test, with * *p* < 0.05, ** *p* < 0.01. Data significance of correlations was determined by Spearman’s test.

In order to identify the role of iron within the tumor, we first stained healthy murine mammary tissue in comparison to both early (8 to 12 weeks) and late (16 to 20 weeks) carcinoma isolated from wildtype PyMT mice applying Perls’ staining (Figure 1a). We observed almost no positive staining in healthy tissue, whereas already in the early carcinoma tissue, a marked increase in positive-stained (blue-colored) areas was noticed. The staining intensity was even more enhanced in late carcinomas, also appearing as brownish staining that might account for hemosiderin deposits (indicated with arrows). We quantified the amount of iron by applying an Image J Macro and showed a significant and progressive increase in iron deposits from early to late-stage carcinoma as well as for the total tumor tissue compared to healthy mammary tissue (Figure 1b). We next asked if these findings correlated with tumor parameters and found a positive, significant correlation of tumor samples with tumor weight (Figure 1c), mean tumor burden (Figure 1d), and the number of affected mammary glands (Figure 1e).

Supporting the concept that the amount of iron in tumors depends on the iron export system via FPN as well as the iron-transporting protein Lcn-2, we aimed at identifying their expression in mammary tumors. Analysis of the TCGA-BRCA (The Cancer Genome Atlas-breast invasive carcinoma) database on the GDC (Genomic Data Commons) data portal of the National Cancer Institute consisting of 110 matched patients (n = 110) showed that there was no difference in mRNA expression of either FPN (Figure 1f) or Lcn-2 (Figure 1g) in breast cancer tissue compared to healthy breast tissue. Along the same line, we did not observe any significant differences regarding the total mRNA expression of FPN (Figure 1h) and Lcn-2 (Figure 1i) in whole tumor homogenates from wildtype PyMT mice compared to healthy murine breast tissue. 

### 2.2. MΦ-Derived Lcn-2 Is Associated with Tumor Progression and Metastasis, Whereas MΦ-Expressed FPN Is Dispensable

**Figure 2:**
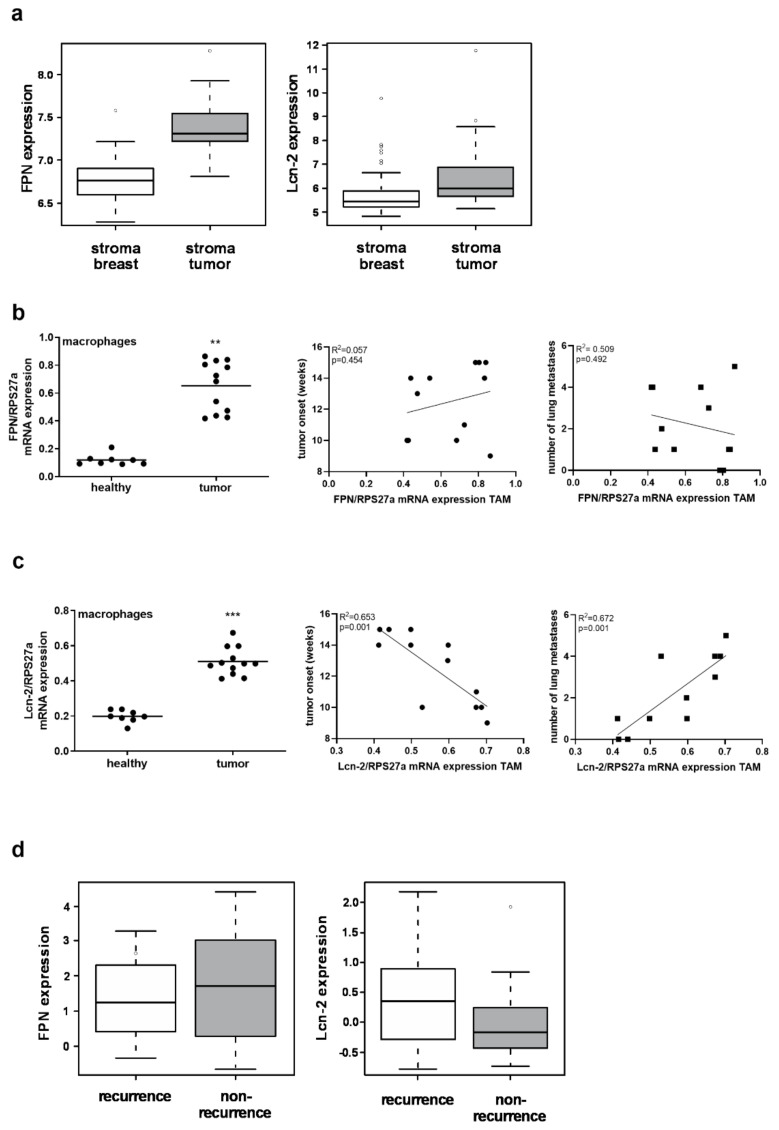
MΦ-derived Lcn-2 is associated with tumor progression. (**a**) Analysis of publicly available GSE files using GNU R for the expression of FPN and Lcn-2 in the tumor stroma compared to the stroma of the healthy breast. (**b**,**c**) Isolation of MΦ from murine PyMT tumors as well as murine healthy breast tissue. High levels of (**b**, left) FPN and (**c**, left) Lcn-2 mRNA expression in tumor-associated macrophages (TAM) compared to MΦ from healthy tissue were observed. Correlation between (**b**, middle and right) FPN and (**c**, middle and right) Lcn-2 mRNA expression to tumor onset (middle) and lung metastasis (right). (**d**) Association of FPN and Lcn-2 expression to recurrence versus non-recurrence in patients in the GSE files with GEO accession codes GSE 1379 (Ma et al. [32]) and GSE 5847 (Boersma et al. [33]). Graphs are displayed as means ± SEM. Statistically significant differences were calculated after analysis of variance (ANOVA) and Bonferroni’s test, with ** *p* < 0.01, *** *p* < 0.001. Data significance of correlations was determined by using Spearman’s rank correlation test.

Taking our previous work regarding the role of MΦ as cellular source of iron within the TME [12,13] and the role of both FPN and Lcn-2 in determining the MΦ iron-release phenotype into account, we aimed at clarifying the role of stromal FPN and Lcn-2 for tumor progression. Data mining of the publicly available GSE data sets using the GEOQuery package for GNU R from the CRAN archive showed that both FPN and Lcn-2 are elevated in tumor stroma compared to the stroma of the healthy breast (Figure 2a). Since MΦ are a major compartment of tumor infiltrates in mammary tumors [28] and considering their pivotal role in iron homeostasis [29,30,31], we isolated MΦ from murine PyMT tumors as well as murine healthy breast tissue. We observed higher levels of both FPN (Figure 2b, left) and Lcn-2 (Figure 2c, left) mRNA expression in tumor MΦ compared to their healthy counterparts. However, correlating FPN (Figure 2b, middle and right) and Lcn-2 (Figure 2c, middle and right) expression to tumor parameters, only Lcn-2 showed a significant correlation to tumor onset (Figure 2c, middle) as well as the number of lung metastases (Figure 2c, right). These findings were verified by evaluating the association of FPN and Lcn-2 to recurrence versus non-recurrence in patients (Figure 2d and as previously reported [12]). GSE files with GEO accession codes GSE 1379 (Ma et al. [32]) and GSE 5847 (Boersma et al. [33]) have been used to analyze the expression of Lcn-2 and FPN. The groups (stroma normal breast and stroma tumor) in GSE 5847 and the groups (recur and non-recur) in GSE 1379 are displayed. 

### 2.3. Extracellular Tumor Fluids of Lcn-2^−/−^ Mice Inhibit Tumor Growth and Metastasis In Vitro

**Figure 3:**
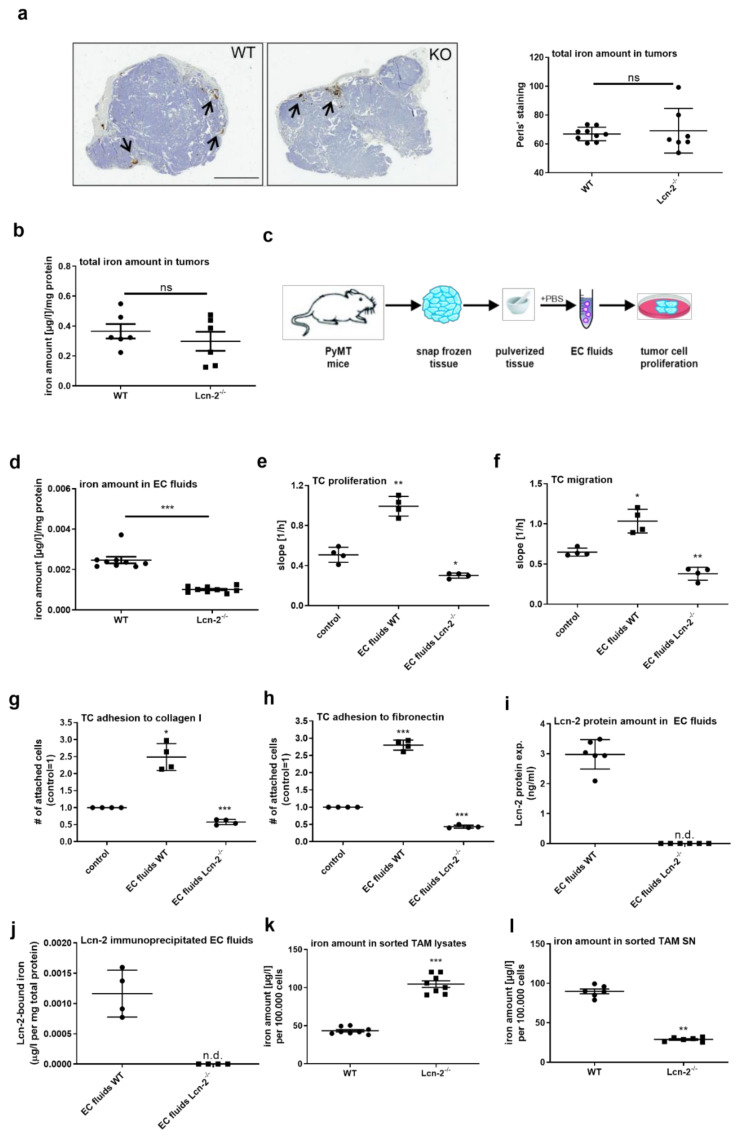
Lcn-2^−/−^ extracellular tumor fluids (EC fluids) inhibit tumor growth and metastasis. (**a**) Determination of the overall iron amount in tumors from WT and Lcn-2^−/−^ (KO) PyMT mice by (**a**, left) Perls’ staining and (**a**, right;) quantification of the Perls’ staining intensity using ImageJ. Scale bar: 6 mm (**b**) Quantitative iron measurement by atomic absorption spectroscopy (AAS). (**c**) Isolation of extracellular fluids from WT and Lcn-2^−/−^ PyMT tumors and (**d**) quantification of the iron content in EC fluids by AAS. (**e**) Effect of EC fluids from WT or Lcn-2^−/−^ tumors on (**e**) cellular proliferation and (**f**) migration of primary WT PyMT tumor cells (TC) using the xCELLigence real-time measurement system. (**g**,**h**) Adhesion assay of PyMT tumor cells (TC) stimulated with WT or Lcn-2^−/−^ EC fluids using collagen I and fibronection as matrix to demonstrate the metastatic potential. (**i**) Analysis of the Lcn-2 protein content in wildtype EC fluids by ELISA. (**j**) Quantification of the Lcn-2-bound iron by AAS after immunoprecipitation of Lcn-2 from EC fluids. (**k**) Intracellular iron amount in TAM isolated from WT and Lcn-2^−/−^ tumors compared to (**l**) the secreted amount of iron in the supernatant. Graphs are displayed as means ± SEM. Statistically significant differences were calculated after analysis of variance (ANOVA) and Bonferroni’s test (**a**,**b**,**d**,**i**–**l**) or Students *t*-test (**e**–**h**), with * *p* < 0.05, ** *p* < 0.01, *** *p* < 0.001. ns: not significant; n.d.: not determined.

Considering the proposed role of Lcn-2 for the availability of iron within the tumor, we determined the overall iron amount in tumors from wildtype (WT) and Lcn-2^−/−^ PyMT mice. Perls’ staining (Figure 3a, left) and the quantification of the Perls’ staining intensity (Figure 3a, right) as well as quantitative iron measurement via atomic absorption spectroscopy (AAS) (Figure 3b) showed no difference between WT and Lcn-2^−/−^ tumors. Given the hypothesis that Lcn-2 is released from MΦ to the TME [12], we isolated extracellular fluids (EC fluids) from both WT and Lcn-2^−/−^ PyMT tumors (Figure 3c). First, we quantified the amount of iron in EC fluids from wildtype and Lcn-2^−/−^ tumors by AAS and found significantly increased iron levels in WT compared to Lcn-2^−/−^ EC fluids (Figure 3d). To evaluate the effect of EC fluids from either WT or Lcn-2^−/−^ tumors on the cellular proliferation (Figure 3e) and migration (Figure 3f) of primary WT PyMT tumor cells, we applied the xCELLigence real-time measurement system. Results showed that only WT EC fluids were able to enhance cellular proliferation and migration, whereas Lcn-2^−/−^ EC fluids showed a negative effect on cellular proliferation and migration. Since collagen I and fibronectin are part of the lung matrix, we performed an adhesion assay of PyMT tumor cells stimulated with either WT or Lcn-2^−/−^ EC fluids to determine the metastatic potential (Figure 3g,h). In line with our previous observations, only WT EC fluids were able to enhance the matrix adhesion of tumor cells, whereas EC fluids from Lcn-2^−/−^ tumors reduced their adhesion, suggesting a reduced metastatic behavior. To prove our hypothesis that Lcn-2 is present in tumor EC fluids, we analyzed the Lcn-2 protein content in wildtype EC fluids by ELISA (Figure 3i) and quantified Lcn-2-bound iron (Figure 3j) via AAS after the immunoprecipitation of Lcn-2 in EC fluids. Both the total Lcn-2 protein amount as well as Lcn-2-bound iron was present in EC fluids isolated from WT PyMT tumors. 

Since TAM may serve as an iron source within the breast tumor microenvironment, we determined the iron amount of TAM isolated from WT and Lcn-2^−/−^ tumors. We observed higher intracellular iron amounts in Lcn-2^−/−^ TAM compared to WT counterparts (Figure 3k). Along these lines, we confirmed less secreted iron in the supernatant of Lcn-2^−/−^ TAM (Figure 3l).

### 2.4. Lcn-2^−/−^ Tumor Cells Show Less Aggressive Tumoral Behavior Due to Reduced Intracellular Iron Amounts

**Figure 4:**
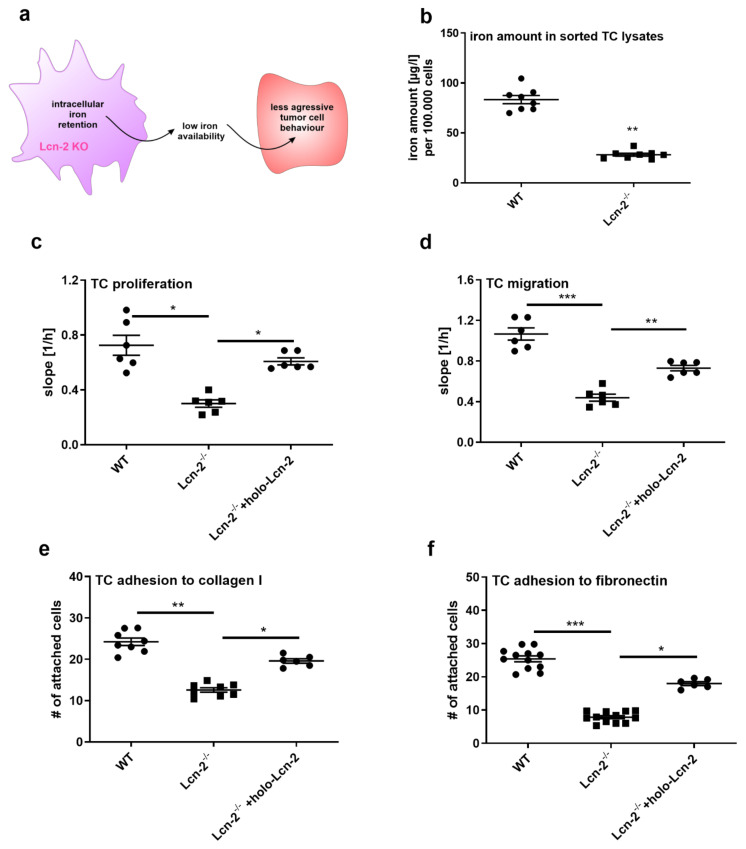
Lcn-2^−/−^ tumor cells are less aggressive and have reduced intracellular iron. (**a**) Tumor cells from Lcn-2^−/−^ (Lcn-2^−/−^ TC) mice show reduced growth and metastatic potential due to lower iron availability in the TME due to iron sequestration in Lcn-2^−/−^ TAM. (**b**) Measurement of the intracellular iron amount in tumor cells (TC) from WT and Lcn-2^−/−^ PyMT tumors. (**c**–**f**) Analysis of the tumor behavior in terms of (**c**) proliferation, (**d**) migration and (**e**,**f**) matrix adhesion, showing a less aggressive tumoral capacity of Lcn-2^−/−^ compared to WT tumor cells (TC). Applying recombinant iron-loaded (holo)-Lcn-2 (1 µg/mL) for 24 h rescued proliferation, migration, and matrix adhesion in Lcn-2^−/−^ tumor cells. Graphs are displayed as means ± SEM. Statistically significant differences were calculated after analysis of variance (ANOVA) and Students *t*-test, with * *p* < 0.05, ** *p* < 0.01, *** *p* < 0.001.

As suggested from our previous studies [34], we speculate that MΦ cells are a major source of available iron within the tumor. Taking into account that MΦ-derived Lcn-2 was significantly correlated to tumor onset and metastasis and the enhanced amount of intracellularly trapped iron within Lcn-2^−/−^ TAM, we further hypothesize that tumor cells from Lcn-2^−/−^ mice show reduced growth and metastatic potential due to lower iron availability in the TME (Figure 4a). First, we measured the actual amount of intracellular iron in tumor cells from WT and Lcn-2^−/−^ PyMT tumors (Figure 4b). Our observations demonstrate a shift of iron availability within tumors: WT MΦ cells have low levels of intracellular iron (Figure 3j), whereas WT tumor cells scavenge high amounts of iron (Figure 4b). In contrast, Lcn-2^−/−^ MΦ cells present an iron-sequestering phenotype with high intracellular iron, while Lcn-2^−/−^ tumor cells have only low iron levels. We next asked if the iron amount in tumor cells reflects their tumoral behavior in terms of cellular proliferation (Figure 4c), migration (Figure 4d), and matrix adhesion (Figure 4e, f). Lcn-2^−/−^ tumor cells present overall less aggressive tumoral capacity than their WT counterparts, showing less proliferation, migration, and matrix adhesion. With regard to the role of iron-loaded Lcn-2 in promoting pro-tumor characteristics, we performed rescue experiments applying recombinant iron-loaded (holo)-Lcn-2 (1 µg/mL, 24 h) to Lcn-2^−/−^ tumor cells, whereby proliferation, migration, and matrix adhesion was induced. 

### 2.5. MΦ-Derived Lcn-2 Promotes Tumor Cell Invasiveness through Its Receptor Lcn-2R in a 3D Spheroid Model In Vitro

**Figure 5:**
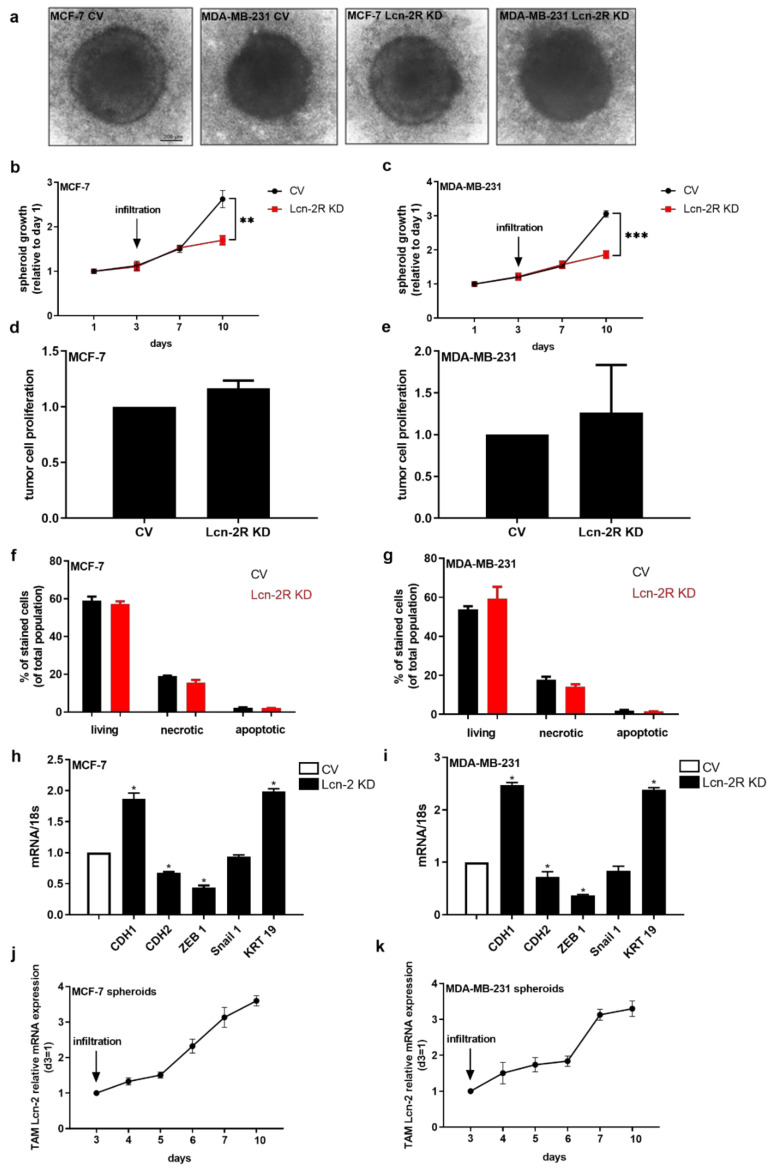
MΦ-derived Lcn-2 promotes tumor cell invasiveness through its receptor Lcn-2R. (**a**) 3D tumor spheroid model using stable Lcn-2R knockdown MCF-7 and MDA-MB-231 breast cancer cells. Representative pictures out of at least four independent experiments are given. In order to mimic the crosstalk of immune cells and tumor cells in the in vitro 3D model, three-day-old tumor spheroids were co-cultured with CD14^+^ immune cells. Scale bar: 200 µm. (**b**,**c**) Spheroid growth of Lcn-2R knockdown (KD) cells after infiltration compared to infiltrated control virus (CV)-transduced cells, measured by the spheroid diameter for each time point. (**d**,**e**) Analysis of proliferative capacity measured by flow cytometry of Ki-67. (**f**,**g**) Measurement of cell death and survival by Annexin V/PI staining. (**h**,**i**) Quantification of EMT marker genes by qRT-PCR for the epithelial markers cadherin (CDH)1 and keratin (KRT)19 and the mesenchymal markers CDH2, ZEB1, and Snail 1 were quantified in tumor cells isolated from infiltrated spheroids. (**j**,**k**) mRNA expression of Lcn-2 in isolated co-cultured MΦ at different time points. Graphs are displayed as means ± SEM. Statistically significant differences were calculated after analysis of variance (ANOVA) and Students *t*-test, with * *p* < 0.05, ** *p* < 0.01, *** *p* < 0.001.

As proof of principle and to substantiate the role of Lcn-2-bound iron in the tumor context, we established a 3D tumor spheroid model using stable Lcn-2R knockdown MCF-7 and MDA-MB-231 breast cancer cells. Knockdown efficiency and maintenance of the knockdown was routinely probed at mRNA (Supplementary Figure S1a,b) and protein level (Supplementary Figure S1c,d). Additionally, we evaluated the basal knockdown effects on tumor spheroid growth to rule out that the knockdown of Lcn-2R had any effect regarding tumor growth per se. Results showed that Lcn-2R knockdown remained without effect regarding tumor cell growth characteristics (Supplementary Figure S2). We found that the knockdown remained stable over the whole period of the experiments and that there was no basal effect of the Lcn-2R knockdown on tumor growth for both cell lines. In order to mimic the crosstalk of immune cells and tumor cells in the in vitro 3D model, we co-cultured three-day-old tumor spheroids with CD14^+^ immune cells (Figure 5a). We observed that there was no difference in spheroid growth during the first four days after infiltration (Figure 5b,c). However, seven days after infiltration, we found a significantly delayed tumor growth of Lcn-2R knockdown (KD) cells compared to control virus (CV)-transduced cells. These differences in growth were neither due to a reduced proliferative capacity (Figure 5d,e), which was measured by flow cytometry analysis of Ki-67, nor enhanced cell death or less survival of the cells (Figure 5f,g), which was analyzed by Annexin V/PI staining. Since we previously described a role for Lcn-2 in promoting epithelial-to-mesenchymal transition (EMT) [24] and taking into account the role of iron for this process [35], we isolated tumor cells from the co-cultured spheroids and analyzed EMT marker genes by real-time qRT-PCR (Figure 5h,i). We observed that Lcn-2R KD cells showed a higher expression of cadherin (CDH)1 and keratin (KRT)19, which are epithelial phenotype markers. In contrast, mRNA expression of CDH2, ZEB1, and Snail 1, which are all markers of the mesenchymal, metastatic phenotype, were significantly reduced in Lcn-2R KD cells. To prove the expression of Lcn-2 in this setting, we isolated co-cultured MΦ at different time points during the co-culture and analyzed the expression of Lcn-2 at mRNA level (Figure 5j,k). We observed a progressively increasing amount of Lcn-2 expression from day 1 to day 3 after tumor spheroid infiltration. Afterwards, Lcn-2 expression levels remained at a constantly high level until day 7 after infiltration. In order to check if MΦ-derived Lcn-2 donates iron to cancer cells, which, in turn, favors tumor growth, we generated MΦ-conditioned medium from either scrambled control scRNA-treated primary human MΦ (scMCM) or Lcn-2-knockdown MΦ (siMCM) and stimulated MCF-7 as well as MDA-MB-231 cells with MCM for 24 h. As a positive control, we applied recombinant iron-loaded holo-Lcn-2 (hLcn-2; 1 µg/mL). We found by AAS measurements that both scMCM- and hLcn-2-treated cancer cells had significantly higher intracellular iron levels compared to siMCM-treated cells (Supplementary Figure S3), thereby underlining our hypothesis. 

## 3. Discussion

In our study, we advance the knowledge on the role of Lcn-2 as a novel iron-transporting protein within the TME. Since MΦ-derived Lcn-2, but not FPN was associated with tumor onset and metastasis in an experimental mammary tumor model as well as with the recurrence status of breast cancer patients, we propose Lcn-2 as a promising candidate influencing the disturbed tumor iron homeostasis. 

The question of how tumor cells acquire iron in order to maintain their malignant growth remains elusive. Major sources of iron within the TME are tumor-infiltrating immune cells such as MΦ that adopt re-wired metabolic activities in order to support tumor growth. In this sense, it is not surprising that tumor-associated immune cells also show a disturbed iron phenotype [12,13,36]. Even if it is known that tumor cells specifically enhance Transferrin (Tf)-bound iron uptake, it might not be exclusive to alternative or additional iron-delivery pathways. With regard to our previous reports [12,23,24], it can be further postulated that Lcn-2 may serve as a new iron-handling protein in MΦ, adopting the macrophage phenotype to the local iron availability. We found that Lcn-2 knockdown MΦ cells polarize towards an iron-sequestering phenotype with impaired iron export functions. Moreover, TAM isolated from Lcn-2^−/−^ PyMT tumors confirmed enhanced iron storage [12], which was correlated with reduced tumor growth and decreased lung metastasis [24]. These observations are further supported by the results from the present study, showing that, upon knockdown of the Lcn-2 receptor, tumor spheroids showed not only reduced tumor growth, but also reduced metastasis-related mesenchymal-state associated genes such as ZEB1 or CDH2, whereas the epithelial markers CDH1 or KRT-19 were significantly enhanced. Still, we observed a decrease in spheroid growth upon Lcn-2R knockdown, which we could not directly attribute to either reduced proliferation or apoptotic or necrotic cell death. We hypothesize that Lcn-2-dependent changes in tumor cell metabolism might account for the observed adaptations in growth. Our own unpublished observations point to the role of Lcn-2-depedendent signaling in promoting cellular stress mechanisms, which, in turn, favor therapy resistance, and thus, tumor growth. Yet, a detailed mechanistic study is still missing and warrants further investigation. 

During recent years, the role of iron bioavailability has been intensively discussed as a potential root cause of malignancy. The accumulation of iron at sites of inflammation, as is the case in tumors, drives the development of reactive oxygen species (ROS), which, in turn, are responsible for malignant transformation. ROS not only rewires cancer cell metabolism towards glycolysis, but also introduces massive modifications in DNA, proteins and lipids, all leading to a more aggressive phenotype. Thus, iron levels are conducive to and necessary for cancer progression. In response to ROS, major antioxidant pathways are induced by the translocation of Nrf2 to the nucleus and downstream target gene induction, which, in turn limits iron accumulation in the labile iron pool by enhancing both iron storage proteins ferritin light (FTL) and heavy (FTH) chain as well as the iron-exporter FPN. Interestingly, it was shown by Song et al. that downregulating Lcn-2 inhibited cell proliferation and induced apoptosis by increasing ROS generation, at least partially via the Nuclear factor erythroid 2-related factor 2 (Nrf2)/heme oxygenase (HO-1) pathway [37]. Since Nrf2 regulates iron efflux from MΦ through the transcriptional regulation of FPN, it was previously suggested that Nrf2 may control the iron metabolic status during inflammation [37,38]. This notion should be expanded to the tumor context, where the inflammatory environment constitutes a major hallmark both during the initiation and progression of tumor development. Furthermore, it was shown that HO-1 activation through heme causes the degradation of the transcription factor Bach-1, which is a transcriptional regulator of FPN [39]. Thus, at least in MΦ, it is possible to control FPN expression independently of hepcidin. Moreover, it might be speculated that either impaired FPN function, additional Lcn-2-induced FPN translational regulatory pathways or Lcn-2-dependent alternative iron-release pathways might take place. A possible explanation for these observations came in a recent publication from Kong et al., reporting that the transcription factor Nrf2 drove FPN transcription through promoter binding and the simultaneous suppression of miR-17-5p [40]. MicroRNAs are small non-coding RNAs that are able to negatively regulate gene expression post-transcriptionally by binding 3′-UTR in target mRNAs [41]. This, in turn, leads to translational repression and/or mRNA degradation. Along these lines, miRNA-20a, a member of the miR-17 seed family, was described to regulate the expression of FPN in lung cancer [42], resulting in iron-sequestration, whereby tumor cell proliferation was favored. Besides the Nrf2 pathway, ROS does also play a major role in activating intracellular signaling pathways, including the EGFR-PI3K-mTOR-c-myc pathway [43]. Interestingly, c-Myc might act as a linker between oncogenic signaling pathways and iron metabolism due to its repressive function on ferritin expression and its activating effect on TfR1 and DMT1 [44]. Intriguingly, patients with increased myc-inducible miR-17-92 showed shorter progression-free survival [45]. Yet, further in-depth studies will be needed to determine the exact mechanistic links and/or interplay between iron export pathways in TAM, especially how Lcn-2 might possibly impact FPN function and/or regulation as well as FPN-independent iron-transport. 

In conclusion, we show that stromal/TAM-derived iron-bound Lcn-2 was linked with breast cancer progression, independent of the expression of the iron exporter FPN. While TAM-derived Lcn-2 was correlated with tumor onset and lung metastasis, TAM-expressed FPN did not associate with any tumor parameter. Interestingly, Lcn-2^−/−^ TAM sequestered iron, which, in turn, favors low iron availability in the TME of Lcn-2^−/−^ PyMT tumors and less tumor growth. However, our study is still lacking mechanistic details following up on a possible negative regulation of FPN in Lcn-2^−/−^ TAM as well as Lcn-2-dependent, FPN-independent iron transport pathways. Moreover, more studies are required to define how Lcn-2-bound iron is recycled within tumor cells and which downstream signaling pathways as well as metabolic signatures are fostered upon the uptake of iron-loaded Lcn-2. 

## 4. Materials and Methods

### 4.1. 3D Tumor Spheroid Experiments

Human MCF-7 and MDA-MB-231 breast cancer cells were obtained from ATCC-LGC Standards GmbH (Wesel, Germany) and were maintained in DMEM medium (Thermo Fisher Scientific, Dreieich, Germany), supplemented with 10% fetal bovine serum (Capricorn Scientific, Ebersdorfergrund, Germany), 100 U/mL penicillin, and 100 μg/mL streptomycin (Sigma-Aldrich, Darmstadt, Germany). Cells were cultivated at 37 °C in a humidified atmosphere with 5% CO_2_. Multicellular spheroids were generated by seeding 10 × 10^3^ breast cancer cells per well in agarose-coated 96-well plates. Cells were subjected to centrifugation at 500× *g* for 4 min and incubated for three days to obtain three-dimensional tumor spheroids. Both control virus-transfected (CV) as well as Lcn-2R-knockdown (Lcn-2R-KD) cells were used to generate 3D spheroids. 

Peripheral blood mononuclear cells (PBMCs) were isolated from human buffy coats (DRK-Blutspendedienst Baden-Württemberg-Hessen, Frankfurt, Germany) using Ficoll separating solution according to the manufacturer’s instructions (Biochrom AG, Berlin, Germany). Subsequently, CD14^+^ monocytes were isolated by magnetic cell sorting using microbeads for human CD14 (Miltenyi Biotec, Bergisch Gladbach, Germany). For spheroid infiltration assays, three-day-old spheroids were co-cultured with 7.5 × 10^4^ CD14^+^ cells for an additional seven days to allow for infiltration and subsequent growth analysis as well as following assays. Spheroid growth was calculated as previously described [24,46,47]. Briefly, we acquired images with a Carl Zeiss Axiovert microscope (Carl Zeiss AG, Jena, Germany) of at least eight spheroids per condition and time point as technical replicates and performed at least four independent experiments. Diameter measurements were determined using AxioVision 40 software (Carl Zeiss AG).

At the end of the experiment, spheroids were washed with PBS, treated with Accutase (Sigma-Aldrich) for 20 min at 37 °C and subjected to a cell strainer (35 μm, Corning, Wiesbaden, Germany) to obtain single cell suspensions. Cells were incubated with microbeads for human CD14 (Miltenyi Biotec), and EpCAM^+^ (CD326, Brilliant Violet421, BD Biosciences, Heidelberg, Germany) tumor cells were isolated by negative selection after magnetic cell sorting. 

### 4.2. Generation of Stable Lcn-2R-Knockdown Cells

A stable Lcn-2R-KD was established in MDA-MB-231 and MCF-7 breast cancer cells. Both breast cancer cell lines were transduced with lentiviruses coding for a non-targeting shRNA or Lcn-2R shRNA (MISSION shRNA, SHCLND-NM_016609, Sigma-Aldrich) followed by puromycin (Sigma-Aldrich) selection to create stable knockdown cell lines.

### 4.3. Flow Cytometry Measurements of 3D Spheroid Experiments

MCF-7 and MDA-MB-231 cells were purchased from ATCC- LGC Standards GmbH and tumor cell spheroids before and after immune cell infiltration were washed with PBS and blocked with 4% FcR Blocking Reagent (Miltenyi Biotec) prior to antibody staining using EpCAM (CD326, Brilliant Violet421, BD Biosciences) and CD45 (Alexa Fluor 700, BD Biosciences). For tumor cell proliferation, cells were stained with Ki-67 (Anti-human Ki-67 APC, Miltenyi Biotec). To discriminate viable from apoptotic and necrotic cells, spheroids were dissociated as described before and stained with FITC-labeled Annexin V (Immunotools, Friesoythe, Germany) and propidium iodide (PI; Fluka, Buchs, Switzerland). Cells were analyzed with a LSRII/Fortessa flow cytometer (BD Biosciences) and analyzed using FlowJo V10 (BD Biosciences).

### 4.4. Tumor Cell Culture of Murine PyMT Breast Cancer Cells

Primary PyMT breast cancer cells were cultured in Dulbecco’s modified Eagle medium (DMEM) with high glucose (Gibco, Dreieich, Germany), supplemented with 100 U/mL penicillin (Sigma-Aldrich, Taufkirchen, Germany), 100 µg/mL streptomycin (Sigma-Aldrich), and 10% fetal bovine serum (FBS; Capricorn Scientific). Tumor cells were cultivated in a humidified atmosphere with 5% CO_2_ at 37 °C and passaged three times per week. Twenty-four hours prior to stimulation, cells were serum starved. Cells were routinely tested for mycoplasma. 

### 4.5. FACS Sorting of Murine TAM and TC

Tissue-derived single-cell suspensions were stained with an antibody cocktail containing CD45 Vioblue (Miltenyi biotec, Bergisch Gladbach, Germany, 130-102-430), CD326 PE (Miltenyi biotec, 130-102-265), F4/80 PE-Cy7 (BD biosciences, Heidelberg, Germany, 123114, Heidelberg, Germany), CD11b Alexa Fluor 700 (BD biosciences, 101257), and 7-AAD (BD biosciences, 559925). CD45^−^/CD326^+^ living tumor cells (TC) and F4/80^+^/CD326^−^ MΦ were sorted using a FACS Aria (BD biosciences). Cells were then transferred to the cell culture and at least cultured for 24 h using Dulbecco’s modified Eagle’s medium (Gibco) for TC culture as described above and RPMI-1640 medium for MΦ, supplemented with penicillin 100 U/mL (Sigma-Aldrich), streptomycin 100 mg/mL (Sigma-Aldrich), and 10% FBS (Capricorn Scientific).

MΦ supernatants were harvested for AAS, whereas MΦ lysates either served for AAS measurements or were used for RNA isolation. RNA isolation and transcription were performed using the RNeasy Micro Kit (Qiagen, Hilden, Germany) and Sensiscript RT Kit (Qiagen) according to the manufacturer’s kit protocols. 

### 4.6. DAB-Enhanced Perls’ Stain and Quantification

Tumor tissue slides were dewaxed in xylene and rehydrated in a series of alcohol solutions using decreasing concentrations. Tumor sections were rehydrated and stained for 10 min with a potassium ferrocyanide/HCl solution (Sigma-Aldrich). After washing in distilled water, slides were treated with 3,3-diaminobenzidinetetrahydrochloride (DAB) (Sigma-Aldrich). Slides were washed again in distilled water, counterstained with Hematoxylin, washed in PBS, dehydrated and mounted using Entellan (Merck, Darmstadt, Germany). Pictures were acquired using the Leica Aperio AT2 S/N 7256 together with the Aperio ImageScope software (Leica, Wetzlar, Germany). The images were analyzed with Fiji [48] and subsequentially quantified for the iron amount by an automatized procedure. The images have been acquired in the custom format using the Leica Aperio AT2 S/N 7256 g system and had to be converted to TIFF files manually. The images were analyzed by employing a custom written ImageJ macro [49]. The images produced by the slide scanner are assumed to be properly white-balanced to be consumable by the ImageJ color deconvolution tool with the H-DAB staining color preset in Fiji [50]. Prior to the color deconvolution of the images, the background was corrected by applying Substract Background in Fiji, which uses the rolling ball algorithm to correct for possible uneven illumination spots and removes possible large spatial variations of the former. Color devolution leads to separate channels and to quantity the fraction of iron; color channel 2 (Color2, DAB channel) was processed with an a priori determined threshold as identified by screening a handful of representatively DAB stained cells. For all remaining cells, the same procedure has to be repeated and thus has been captured in an ImageJ macro for repeated application.

### 4.7. Generation of EC Fluids

Frozen tumor tissues from either wildtype of Lcn-2^−/−^ PyMT tumors were crushed into fragments < 2 mm in diameter and suspended in 1:2 weight/volume of 2x phosphate-buffered saline (PBS). The solution was rotated at 4 °C for 3 h. The samples were then vortexed, and the centrifugation-cleared supernatants were used for experiments.

### 4.8. Atomic Absorption Spectrometry 

The iron content of TAM- and TC-lysates as well as TAM supernatants and EC fluids were determined by graphite furnace atomic absorption spectrometry (AAS). Samples were measured as triplicates with a PinAAcleTM 900 T atomic absorption spectrometer (PerkinElmer, Rodgau, Germany). Slit 0.2 nm and wavelength 248.33 nm were used as spectrometer parameters. A hollow cathode iron lamp (30 mA maximum operating current) was run at 100% maximum current. The calibration solutions (10 µg/L to 90 µg/L) were prepared by adequate dilution of iron standard for AAS (Sigma-Aldrich) stock solution. A pyrolysis temperature of 1400 °C and an atomization temperature of 2100 °C were used.

### 4.9. Quantitative Real-Time PCR (qRT-PCR)

Gene expression profiles were determined by qPCR using the SYBR Green Supermix (Bio-Rad, Munich, Germany) on a CFX-Connect real-time-PCR detection system (Bio-Rad). Results were quantified using the Bio-Rad CFX-Manager (Bio-Rad, version 3) with RPS27a mRNA expression as a housekeeping control. All primers were purchased from Biomers (Ulm, Germany):

Lcn-2 (Fw: 5-CTCACCACTCGAGGTA-3; Rev: 5-GACCCGCAAATATGCC-3)
FPN (Fw: 5-TGAGCCTCCCAAACCGCTTCCATA-3; Rev: 5-GGGCAAAAAGA-TACAACGACGACTT-3)
ZEB1 (Fw: 5-CCGCCCGAGTTCCAGTGGTA-3; Rev: 5-GGCCTCCTTCTCATGTATCTGGG-3)
KRT19 (Fw: 5-GATCGAAGGCCTGAAGGAAGAG-3; Rev: 5-AATCCACCTCCACACTGACCTG)
CDH1 (Fw: 5-TTCCTCCCAATACATCTCCC-3; Rev: 5-TTGATTTTGTAGTCACCCACC-3)
CDH2 (Fw: 5-CCTGGAGACATTGGGGACTTCA-3; Rev: 5-GCCACTGCCTTCATAGTCAAACAC-3)
RPS27a (Fw: 5-GACCCTTACGGGGAAAACCAT-3; Rev: 5-AGACAAAGTCCGGCCATCTTC-3).

### 4.10. Migration Assay

The RTCA DP xCELLigence instrument (OLS, Bremen, Germany) was used to measure the migration of cells. Cells were added into the upper chamber of a two-chamber CIM-plate (OLS) and stimulated with EC fluids from either wildtype or Lcn-2^−/−^ tumors. Where indicated, cells were co-stimulated with holo-Lcn-2 (1 µg/mL). Continuous recording of migration was accomplished for 24 h. Data are displayed as a measure for time-dependent impedance changes, which is represented as the normalized slope per hour (slope 1/h) of the normalized cell index. The RTCA Software 1.2 (OLS) was used for acquisition and analysis.

### 4.11. Proliferation Assay

Proliferation of primary tumor-derived cancer cells was measured using the RTCA DP xCELLigence instrument (OLS) as described previously [23]. Cells were stimulated with EC fluids from either wildtype or Lcn-2^−/−^ tumors. Where indicated, cells were co-stimulated with holo-Lcn-2 (1 µg/mL). Continuous recording of the proliferation was accomplished for 72 h and data are displayed as time-dependent impedance changes, represented as the normalized slope per hour (slope 1/h) of the normalized cell index. The RTCA Software 1.2 (OLS) was used for acquisition and analysis.

### 4.12. Adhesion Assay

Cells were stimulated with EC fluids from either wildtype or Lcn-2^−/−^ tumors for 24 h, with the rescue of Lcn-2-dependent effects by addition of holo-Lcn-2 (1 µg/mL) and marked with cell tracker green (Thermo Fisher Scientific). Afterwards, cells were counted and seeded onto collagen I (10 µg/mL; Thermo Fisher Scientific) or fibronectin (10 µg/mL; Sigma-Aldrich) pre-coated wells for 2 h, washed, and fixed with 4% PFA. Five pictures were taken from each group from at least three independent experiments using triplicates. The number of attached cells was determined using the ImageJ automated macro-based analysis (National Institutes of Health, Bethesda, MD, USA).

### 4.13. Lcn-2 ELISA

EC fluids were isolated as described above. A volume of 100 µl of each sample was applied to an ELISA well-plate previously covered with the anti-Lcn-2 antibody (R&D Systems, Wiesbaden, Germany) and blocked for 1 h. After sample incubation, the detection anti-Lcn-2 antibody was added. HRP-conjugated avidin (Thermo Fisher Scientific) was incubated for 1 h, the color reagent (OPD tablets; Agilent, Frankfurt, Germany) was added, and color development was assessed.

### 4.14. Lcn-2 Immunoprecipitation

For immunoprecipitation (IP), EC fluids from both healthy murine mammary tissue and wildtype PyMT tumor tissue were isolated. Dynabeads (Thermo Fisher Scientific) were added and incubated overnight at 4 °C in the presence of a specific antibody against Lcn-2 (R&D systems). Beads were precipitated using the DynaMag-2 magnet (Thermo Fisher Scientific) and washed three times with IP buffer. Protein was eluted by addition of 2x loading buffer and incubated at 95 °C for 5 min. 

### 4.15. Generation of Recombinant Lcn-2

Recombinant human Lcn-2 was produced by transformation of *E. coli* with a pGEX-4T-3-NGAL plasmid as already described [24]. In order to test efficient Lcn-2-catechol-iron complex formation, UV-visible spectroscopy (UV-vis) was routinely used. Therefore, 10 µM Lcn-2 were incubated with 10 µM catechol (Sigma-Aldrich) and 10 µM iron (Sigma-Aldrich).

### 4.16. MMTV-PyMT Breast Cancer Model

Wildtype and Lcn-2^−/−^ mice expressing the Polyoma-middle-T oncogene (PyMT) in the C57BL/6 background were used. For genotyping, tail-tips were lysed with KAPA Genotyping lysis buffer (Peqlab, Erlangen, Germany) and the resulting DNA solutions were analyzed by PCR amplification using KAPA Hotstart Genotyping reaction mix (Peqlab). Early-stage tumors were harvested at weeks 8 to 12; late-stage tumors were harvested at weeks 16 to 20. 

### 4.17. GSE Files

The given data were assessed with gene expression profiles from accessible microarray data sets for FPN and Lcn-2. Relevant Gene Expression Omnibus (GEO) records have been identified, based on the corresponding publications, as stored in the NCBI data base [51] and the associated Series data sets (GSE files) with GEO accession codes GSE 1379 (Ma et al. [32]) and GSE 5847 (Boersma et al. [33]) have subsequently been retrieved and analyzed with GNU R [52] and the package GEOquery [53]. To this end, the expression data for LCN-2 and SLC40A1 have been extracted from all samples (GSM files) from the GSE file and were annotation-filtered for the required groups. Statistical analyses have been conducted with GNU R [52]. The groups (Stroma normal breast and stroma tumor) in GSE 5847 and the groups (recur and non-recur) in GSE 1379 each have been tested to be in accordance with a normal distribution by means of the Shapiro-Wilk test. If the null hypothesis (normally distributed) was not rejected, then an F-Test for variance homoscedasticity was used, otherwise the Levene Test was used. Based on the outcome of the latter test, i.e., the data have been identified to be heteroscedastic, then the two-sided Welch *t*-test was used to assess if there were differences between the groups, otherwise the two-sided classical Student’s *t*-test was used. 

### 4.18. TCGA Data Base Analysis

To show the mRNA expression of FPN and Lcn-2 in breast tumors, gene expression data of the Cancer Genome Atlas were analyzed (https://portal.gdc.cancer.gov/, last accessed data: 29 April 2020). Expression data of the TCGA-BRCA project was used. Available patients with expression data in breast cancer tissue as well as corresponding healthy tissue were sub-selected and log 2 normalized.

### 4.19. Statistical Analyses

Statistical analyses were performed applying GraphPad Prism^®^ 8 software (GraphPad Software, San Diego, CA 92108, USA). The distribution of variables was tested for normality using the Kolmogorov–Smirnov test. Accordingly, statistically significant differences were calculated after analysis of variance (ANOVA) and Bonferroni’s test or Students *t*-test. Data significance of correlations was determined by Spearman’s test including all investigated groups. Data are expressed as means ± SEM. P values were considered significant at * *p* < 0.05, ** *p* < 0.01, *** *p* < 0.001.

## Data Availability

Data available in a publicly accessible repository that does not issue DOIs. Publicly available datasets were analyzed in this study. This data can be found here: GEO accession codes GSE 1379 (Ma et al. [32]) and GSE 5847 (Boersma et al. [33]).

## Supplementary Materials

The following are available online at https://www.mdpi.com/2218-1989/11/3/180/s1; Figure S1: 3D tumor spheroid model using stable Lcn-2R knockdown MCF-7 and MDA-MB-231 breast cancer cells. Knockdown efficiency and maintenance of the knockdown was routinely probed at mRNA (Supplementary Figure S1a,b) and protein level (Supplementary Figure S1c,d), Figure S2: Basal knockdown effects on tumor spheroid growth, Figure S3: Iron levels of MCM-treated tumor cells. Supplemental material and methods: Generation of MΦ-conditioned medium (MCM) and MCM-stimulation of breast cancer cells. Supplemental references.
