# Supplementary material for: Iron-Bound Lipocalin-2 from Tumor-Associated Macrophages Drives Breast Cancer Progression Independent of Ferroportin
Source: Metabolites. 2021 Mar 19;11(3):180. doi: 10.3390/metabo11030180 (PMC8003561; doi:10.3390/metabo11030180)
Supplement: Supplementary file 1 [file metabolites-11-00180-s001.pdf]

metabolites

MDPI

Supplemental Information

Iron-bound lipocalin-2 from tumor-associated macrophages drives breast cancer progression independent of ferroportin

Christina Mertens[1], Matthias Schnetz[1], Claudia Rehwald[1], Stephan Grein[2], Eiman Elwakeel[1], Andreas Weigert[1,3], Bernhard Brüne[1,3], Michaela Jung[1]*

Figure S1*: 3D tumor spheroid model using stable Lcn-2R knockdown MCF-7 and MDA-MB-231 breast cancer cells. Knockdown efficiency and maintenance of the knockdown was routinely probed at mRNA (Suppl. Fig. S1a, b) and protein level (Suppl. Fig. S1c, d)*

Figure S2*: Basal knockdown effects on tumor spheroid growth*

Figure S3*: Iron levels of MCM-treated tumor cells*

Supplemental material and methods: *Generation of MΦ-conditioned medium (MCM) and MCM-stimulation of breast cancer cells*

Supplemental references

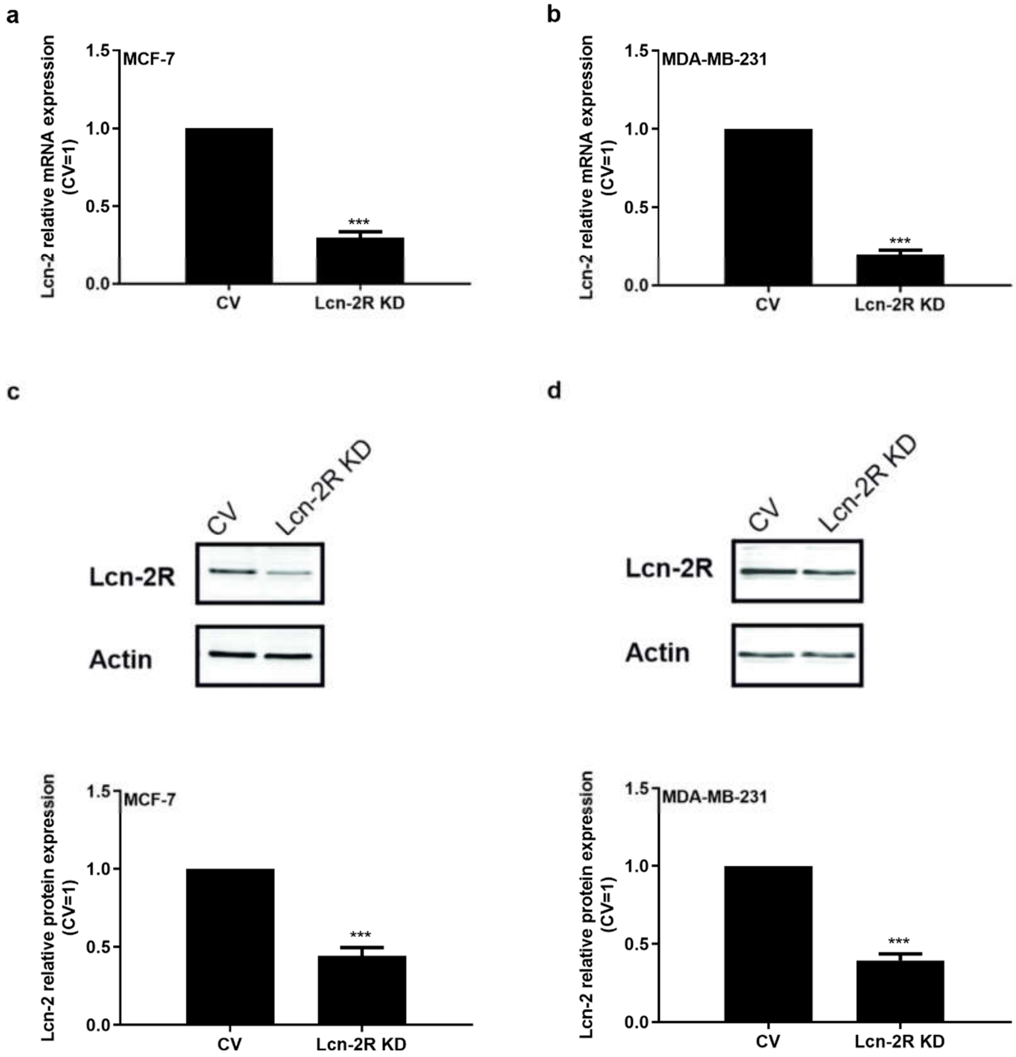

Supplemental Figure S1. *3D tumor spheroid model using stable Lcn-2R knockdown MCF-7 and MDA-MB-231 breast cancer cells. (a-d) Knockdown efficiency and maintenance of the knockdown was routinely probed at (a, b) mRNA and (c, d) protein level. Graphs are displayed as means ± SEM. Statistically significant differences were calculated after analysis of variance (ANOVA) and Students t-test, with ***p < 0.001.*

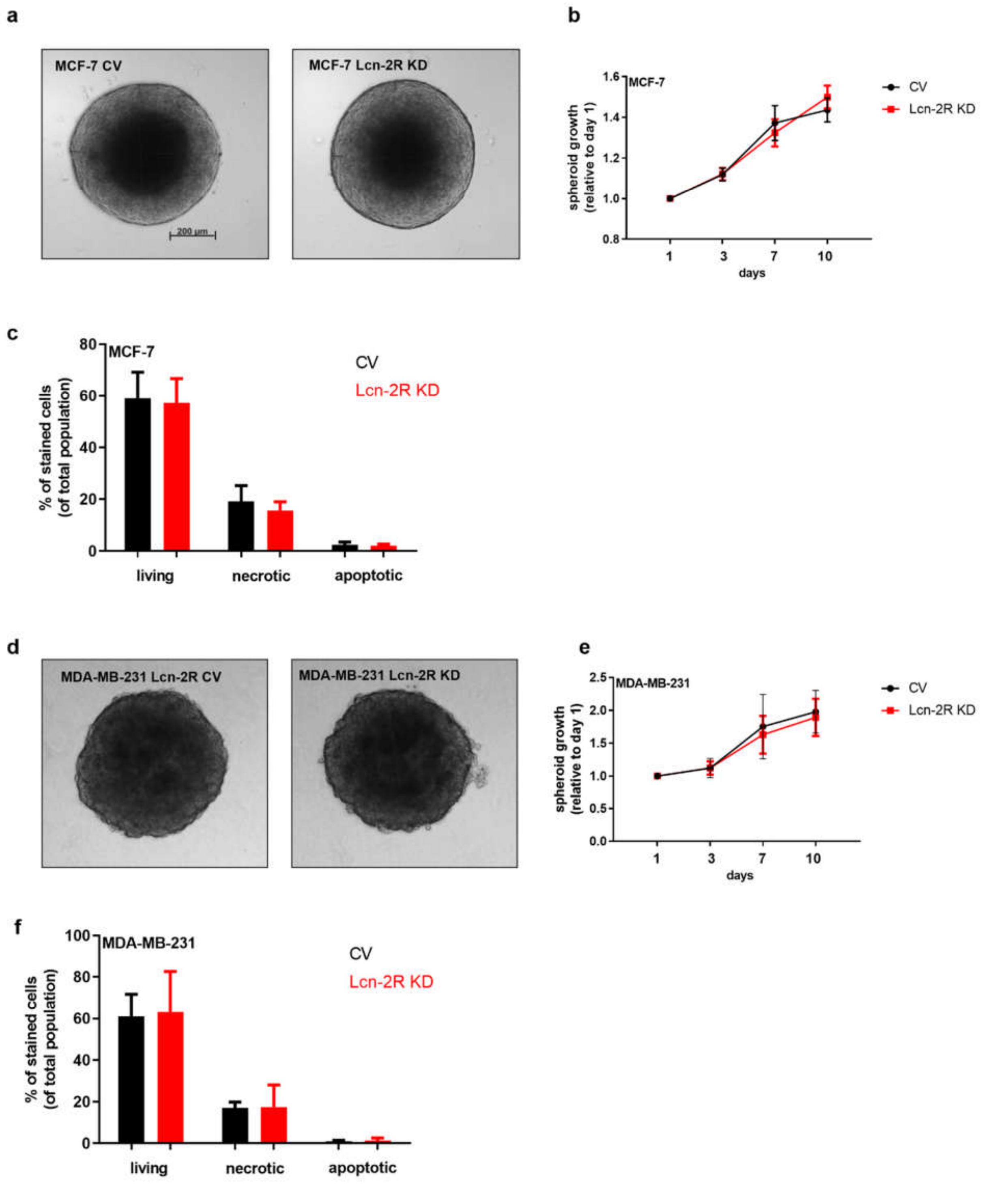

Supplemental Figure S2. *Basal knockdown effects on tumor spheroid growth. (a) 3D tumor spheroid model using stable Lcn-2R knockdown MCF-7 and MDA-MB-231 breast cancer cells. Representative pictures out of at least 4 independent experiments are given. (b, c) Spheroid growth of Lcn-2R knockdown (KD) cells compared to infiltrated control virus (CV)-transduced cells, measured by the spheroid diameter for each time-point. (d, e) Measurement of cell death and survival by Annexin V/PI staining. Graphs are displayed as means ± SEM. Statistically significant differences were calculated after analysis of variance (ANOVA) and Students t-test.*

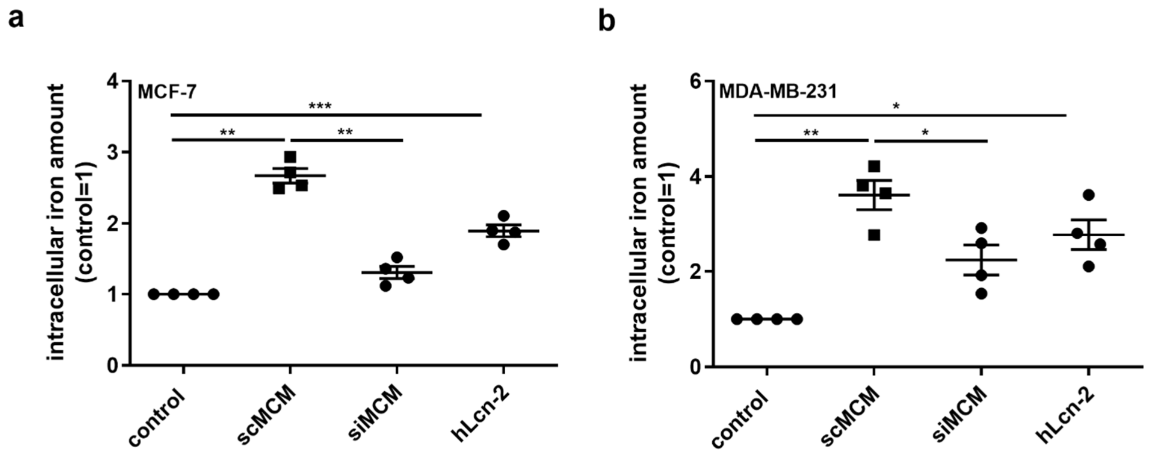

Supplemental Figure S3. *Iron levels of MCM-treated tumor cells. (a) MCF-7 and (b) MDA-MB-231 breast cancer cells were stimulated with* MΦ*-conditioned medium (MCM) from scRNA-treated control* MΦ *or Lcn-2-knockdown* MΦ *(siMCM) for 24 h. Supernatant of unstimulated MΦ served as control. Recombinant iron-loaded Lcn-2 (1 µg/ml) served as positive control. Iron levels were measured by AAS and are represented as relative iron levels with respect to untreated control cells. Graphs are displayed as means ± SEM. Statistically significant differences were calculated after analysis of variance (ANOVA) and Students t-test, with *p < 0.05, **p < 0.01, ***p < 0.001.*

Supplemental material and methods

Generation of MΦ-conditioned medium (MCM) and MCM-stimulation of breast cancer cells

Human monocytes were isolated from commercially available buffy coats (DRK-Blutspendedienst Baden-Württemberg-Hessen, Frankfurt, Germany) using Ficoll-Hypaque gradients (PAA Laboratories, Solingen, Germany). Monocytes were differentiated into primary human MΦ with RPMI 1640 containing 5% AB-positive human serum (DRK-Blutspendedienst) for 7 days and achieved approximately 80% confluence.

GenMute® transfection reagent (SignaGen, Offenbach, Germany) was used according to the manufacturer's instructions to knockdown Lcn-2. 50 nM LCN-2 siRNA (Qiagen, Hilden, Germany) was transfected into primary human MΦ. A non-targeting, scrambled siRNA (sictrl; Qiagen) was used as control.

24 h prior to stimulation, cells were serum-starved. According to our previous studies [1, 2, see below], 20 ng/ml IL-10 (Peprotech, Hamburg, Germany) was used for 24 h to induce an iron-release MΦ phenotype with enhanced secretion of iron-loaded Lcn-2. Conditioned media of polarized MΦ (MCM) were collected, centrifuged at 1000 x g for 5 min at 4°C, and aliquots were stored at -80°C until further use. Supernatant of unstimulated MΦ served as control.

Human MCF-7 and MDA-MB-231 breast cancer cells were maintained in DMEM medium (Thermo Fisher Scientific, Dreieich, Germany), supplemented with 10% fetal bovine serum (Capricorn Scientific, Ebersdorfergrund, Germany), 100 U/ml penicillin, and 100 µg/ml streptomycin (Sigma-Aldrich, Darmstadt, Germany). Cells were cultivated at 37°C in a humidified atmosphere with 5% CO_2. 24 h prior to stimulation, cells were serum-starved. We stimulated breast cancer cells with MCM from scRNA-treated MΦ (scMCM) or siLcn-2- MΦ (siMCM) for 24 h. Supernatants from unstimulated MΦ served as control. Treatment with recombinant iron-loaded Lcn-2 (hLcn-2; 1 µg/ml) served as positive control. Afterwards, cells were washed and harvested and the cell lysates were used to quantify intracellular iron levels via AAS measurements.

Supplemental References:

1. Schnetz M, Meier JK, Rehwald C, Mertens C, Urbschat A, Tomat E, Akam EA, Baer P, Roos FC, Brune B *et al*: **The Disturbed Iron Phenotype of Tumor Cells and Macrophages in Renal Cell Carcinoma Influences Tumor Growth**. *Cancers (Basel)* 2020, **12**(3).
2. Mertens C, Akam EA, Rehwald C, Brune B, Tomat E, Jung M: **Intracellular Iron Chelation Modulates the Macrophage Iron Phenotype with Consequences on Tumor Progression**. *PLoS One* 2016, **11**(11):e0166164.
